# The Draft Genome of the “Golden Tide” Seaweed, *Sargassum horneri:* Characterization and Comparative Analysis

**DOI:** 10.3390/genes14101969

**Published:** 2023-10-21

**Authors:** Shengqin Wang, Mingjiang Wu

**Affiliations:** 1 National and Local Joint Engineering Research Center of Ecological Treatment Technology for Urban Water Pollution, Wenzhou University, Wenzhou 325035, China; mysango@163.com; 2 Zhejiang Provincial Key Laboratory for Subtropical Water Environment and Marine Biological Resources Protection, Wenzhou University, Wenzhou 325035, China; 3 College of Life and Environmental Science, Wenzhou University, Wenzhou 325035, China

**Keywords:** *Sargassum horneri*, golden tide, genome, next-generation sequencing

## Abstract

*Sargassum horneri*, a prevalent species of brown algae found along the coast of the northwest Pacific Ocean, holds significant importance as a valuable source of bioactive compounds. However, its rapid growth can lead to the formation of a destructive “golden tide”, causing severe damage to the local economy and coastal ecosystems. In this study, we carried out de novo whole-genome sequencing of *S. horneri* using next-generation sequencing to unravel the genetic information of this alga. By utilizing a reference-guided de novo assembly pipeline with a closely related species, we successfully established a final assembled genome with a total length of 385 Mb. Repetitive sequences made up approximately 30.6% of this genome. Among the identified putative genes, around 87.03% showed homology with entries in the NCBI non-redundant protein database, with *Ectocarpus siliculosus* being the most closely related species for approximately one-third of these genes. One gene encoding an alkaline phosphatase family protein was found to exhibit positive selection, which could give a clue for the formation of *S. horneri* golden tides. Additionally, we characterized putative genes involved in fucoidan biosynthesis metabolism, a significant pathway in *S. horneri*. This study represents the first genome-wide characterization of a *S. horneri* species, providing crucial insights for future investigations, such as ecological genomic analyses.

## 1. Introduction

*Sargassum horneri* is a major brown macroalga commonly found along the northwest coast of the Pacific Ocean [1]. Since its discovery in the eastern Pacific in 2003, the range of this macroalga has rapidly expanded [2]. Typically inhabiting the sub-tidal zone, *S. horneri* thrives at depths ranging from 3 to 15 m. It plays a crucial role in carbon cycling by effectively sequestering substantial amounts of CO_2_, thus holding significant importance in marine ecosystems [3]. Extensive research has highlighted *S. horneri* as a valuable source of bioactive compounds, particularly sulfated polysaccharides, which exhibit a wide range of functions, including antiviral and anti-inflammatory activities [4,5]. For instance, fucoidan derived from *Sargassum* has shown significant efficacy in reducing *Helicobacter pylori* infection without inducing drug resistance [6]. The potential of *S. horneri* as a source of bioactive compounds makes it a subject of great interest for further research and exploration. Understanding and harnessing the properties of *S. horneri* could have implications for environmental conservation and the development of novel pharmaceuticals.

It is important to note that *S. horneri* can also pose significant challenges to coastal ecosystems and local economies. The phenomenon of drifting *S. horneri*, driven by heightened light energy compared to its benthic state, can result in rapid growth and the formation of what is commonly known as a “golden tide” [7,8]. This proliferation can result in severe damage to coastal ecosystems and have negative repercussions on local economies. In the Yellow Sea, a *S. horneri* golden tide hazard caused an economic loss of about CNY 0.5 billion due to damage to local seaweed aquaculture [9]. In recent years, there has been a significant increase in the occurrence and distribution of the “golden tide” seaweed phenomenon. Coastal eutrophication is a potential factor driving the explosive growth of *S. horneri* [10]. Nitrogen-enriched treatments have been found to significantly increase the growth rate of S. horneri under laboratory conditions [11]. Phosphorus is also another important nutrient that can cause increased growth of algae [12]. However, the molecular mechanisms underlying the phenomenon known as the “golden tide” have not been thoroughly elucidated.

Brown algae, which consists of approximately 2000 species and are categorized into 16 orders, are a prominent group of multicellular organisms that can be found extensively in marine environments [13]. There are only a few brown algae genomes that have been sequenced, including *Ectocarpus siliculosus* [14], *Saccharina japonica* [15], *Nemacystus decipiens* [16], *Cladosiphon okamuranus* [17], *Tribonema minus* [18], *Macrocystis pyrifera* [19], *Sargassum fusiforme* [20], and *Undaria pinnatifida* [21,22]. These genome sequences were collected in June 2022. However, the ongoing project Phaeoexplorer is expected to provide dozens more genomes in the future. At present, there is a shortage of genomic resources for *S. horneri* in public databases. However, the genome sequence of *S. fusiforme* has been successfully decoded, making it the first *Sargassum* genus genome to be assembled using a combination of PacBio and Ilumina reads. With a total length of approximately 394.4 MB and an N50 value of around 142.1 KB, the assembled genome of *S. fusiforme* is highly complete, with over 90% of the BUSCOs detected at the protein level. As such, it provides an invaluable reference for guiding the genome sequencing process of *S. horneri*. Incorporating comparative information during the assembly process can significantly enhance the quality of the reconstructed genome. 

This research aimed to characterize the genome of *S. horneri* and generate molecular resources for studying the “golden tide” seaweed. To achieve this, we conducted de novo whole-genome sequencing of a strain of *S. horneri* using the Illumina HiSeq X-ten platform. The genome assembly was performed using a reference-guided assembly pipeline, followed by further analysis, including the characterization of genome features, identification of repetitive sequences, gene prediction, and comparative analysis. Additionally, we discovered and examined genes responsible for the regulation of fucoidan biosynthesis enzymes. 

## 2. Materials and Methods

### 2.1. Materials and DNA Extraction

The *S. horneri* strain was collected from the sea surface in the Wenzhou Dongtou District of Zhejiang Province, China. It underwent cleaning and disinfection using ddH_2_O, followed by freezing and storage in liquid nitrogen for genomic DNA extraction. Genomic DNA extraction was carried out using a plant genomic DNA extraction kit obtained from Annoroad Gene Technology in Beijing, China. Subsequently, the extracted DNA was quantified and assessed for quality using a NanoDrop 2000 microspectrophotometer, a Qubit fluorometer, and 1% agarose gel electrophoresis in same company.

### 2.2. Genome Sequencing, Assembly, and Characterization

A paired-end (PE) library was created following the Illumina standard protocol, with insert sizes of 350 base pairs (bps). The raw sequence data, consisting of reads with a length of 150 bp, were generated using an Illumina HiSeq X-ten platform. The entire set of sequencing reads was deposited in the Short Read Archive (SRA) database under the accession number PRJNA756794. After removing the adapters, the fastp program was employed to filter out reads containing more than 10% of N nucleotides or more than 20% of low-quality bases [23]. The resulting clean reads were then utilized to assess genome size, the proportion of repetitive sequences, and heterozygosity. The k-mer count distribution (k = 21) was calculated using Jellyfish v2.2.10 [24], and Genomescope was employed for genome size estimation [25]. Next, the clean reads were assembled into contigs and scaffolds using a reference-guided de novo assembly pipeline [26], which has been successfully used to construct the *Cardamine amara* genome [27]. First, quality trimmed paired-end reads were checked using FastQC V0.11.9, and then mapped to the genome sequence of *S. fusiforme* using the bowtie2 V2.3.5.1 with the default parameters. Blocks with continuous read coverage were obtained and nearby blocks were combined to form a superblock, which was then subjected to ABYSSS for de novo assembly. The scaffolds were subjected to redundancy removal and error correction by aligning the trimmed paired-end reads. Uncovered regions of the contigs were eliminated, and the remaining contigs were split. Contigs with a length shorter than 200 bp were discarded, and scaffolds shorter than 1 kb were also removed from further analysis. Finally, contigs were aligned to the Nt database using an E-value threshold of 1 × 10^−5^, and the top 10 matches to eukaryotes were filtered out. To assess the accuracy of the genome assembly, the clean reads were realigned to the assembly using bowtie2. 

To analyze the distribution of repetitive sequences within the assembled genome, we consolidated the genome into a singular sequence. This was achieved by inserting 100 “N” characters between each pair of linked contigs. Afterwards, Repeat-Modeler was utilized to construct a de novo repeat library for *S. fusiforme* [28]. This process involved the integration of three repeat-finding methods: RECON [29], RepeatScout [30], and TRF [31]. Subsequently, RepeatMasker V4.0.9 was employed, using the generated repeat library as input and the default search engine rmblast, to identify potential homologous repeats within the assembled genome. Finally, the repeat-masked genome was used for further analysis. 

### 2.3. Gene Prediction and Functional Annotation

Genome annotation was conducted using the BRAKER1 pipeline, which combines the strengths of GeneMark-ET and AUGUSTUS [32]. To begin, RNA-Seq data of *S. horneri* (PRJDB4109) were obtained from NCBI and aligned to the repeat-masked assembly using TopHat2 V2.1.1 [33]. The resulting alignment files, along with transcript data, were utilized to generate initial gene structures using the GeneMark-ET [34]. AUGUSTUS was used to further train these initial gene structures, resulting in the final gene predictions [35]. To assess the completeness of the putative genes, BUSCO V3.0.2 and the eukaryota_odb9 database were employed. 

Functional annotations for the putative genes were performed by comparing their sequences with public databases, including the NCBI non-redundant protein database (Nr), Swiss-Prot, and Pfam. Gene Ontology (GO) terms and COG were annotated using eggNOG-mapper V2 using the default parameters [36]. In addition, the KAAS web service was used to map the putative *S. horneri* genes onto the KEGG metabolic pathways. Genes from other plant species, such as *Arabidopsis thaliana*, *Chlamydomonas reinhardtii*, *Monoraphidium neglectum*, *Auxenochlorella protothecoides*, *Ostreococcus lucimarinus*, *Ostreococcus tauri*, *Micromonas commoda*, *Micromonas pusilla*, *Cyanidioschyzon merolae*, *Galdieria sulphuraria*, and *Chondrus crispus* (carragheen), were included in the analysis. 

Given the significance of fucoidan in the *Sargassum* genus, we embarked on an extensive study to identify and characterize the specific genes associated with the biosynthesis and metabolism of fucoidan. The synthesis of fucoidan is likely catalyzed by six enzymes, including GDP-mannose 4,6-dehydratase (GM46D), GDP-l-fucose synthetase (GFS), L-fucokinase (FK), GDP-fucose pyrophosphorylase (GFPP), fucosyltransferase (FT), and sulfotransferase (ST). Genes encoding these enzymes were manually confirmed based on the Blast search results against the Nr database.

### 2.4. Comparative Analysis

**Figure 1:**
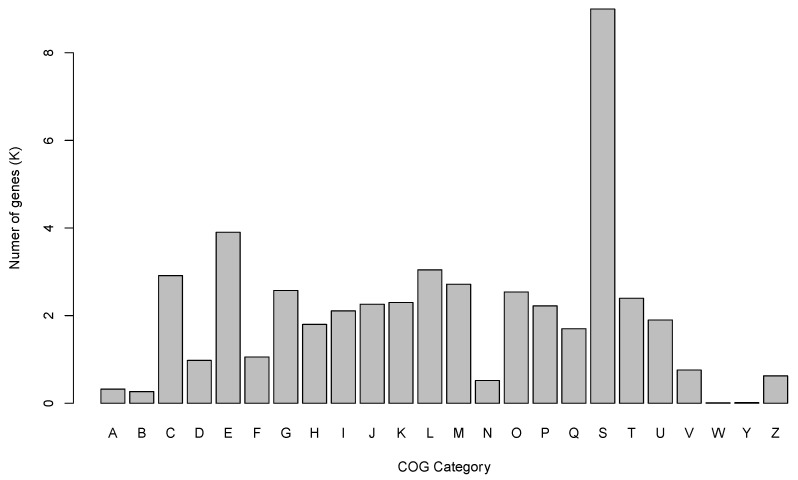
COG functional classification of putative genes in the S. *horneri* genome: [A] RNA processing and modification; [B] chromatin structure and dynamics; [C] energy production and conversion; [D] cell cycle control, cell division, chromosome partitioning; [E] amino acid transport and metabolism; [F] nucleotide transport and metabolism; [G] carbohydrate transport and metabolism; [H] coenzyme transport and metabolism; [I] lipid transport and metabolism; [J] translation, ribosomal structure and biogenesis; [K] transcription; [L] replication; recombination and repair; [M] cell wall/membrane/envelope biogenesis; [N] cell motility; [O] post-translational modification, protein turnover, and chaperones; [P] inorganic ion transport and metabolism; [Q] secondary metabolites biosynthesis, transport, and catabolism; [S] function unknown; [T] signal transduction mechanisms; [U] intracellular trafficking, secretion, and vesicular transport; [V] defense mechanisms; [W] extracellular structures; [X] COG not assigned; [Y] nuclear structure; [Z] cytoskeleton.

Ortholog analysis was performed using OrthoMCL V2.0.9 and protein datasets obtained through the BRAKER1 pipeline. In addition to the *S. horneri* dataset, the analysis included eight other brown algae species: *E. siliculosus*, *S. japonica*, *M. pyrifera*, *N. decipiens*, *C. okamuranus*, *T. minus*, *S. fusiforme*, and *U. pinnatifida*. For *S. japonica*, transcriptome sequencing data from different tissue sites (SRA accession numbers: SRX5192067- SRX5192070) were used, while RNA-seq datasets for *M. pyrifera* from different genders (SRA accession numbers: SRX2352371, SRX2352374) were utilized in the BRAKER1 pipeline. The data for the other species were directly download from the official website. In each organism, CD-HIT was employed to eliminate redundant sequences with a similarity of 90% or higher. Following this, the non-redundant protein sequences underwent an all-against-all Blastp analysis, utilizing an E-value threshold of 1 × 10^−5^. By employing OrthoMCL and examining the intersections of orthologs across the seven brown algae, a total of 14,819 groups were identified, as illustrated in Figure 1 [D]. Subsequently, from these groups, 2287 were recognized as one-to-one orthologs across the species. The protein sequences were aligned using MAFFT V7.470 [37], and the resulting alignments were then trimmed using trimAl V1.4.rev15 [38]. The trimming procedure employed the heuristic “automated1” method to select the optimal approach. After the alignments were trimmed, they were concatenated, and a phylogenetic tree was constructed using RAxML (v8.2.12) with the PROTGAMMALG model [39]. The constructed phylogenetic tree was visualized using iTOL [40].

The PosiGene pipeline was used to search the genome for positively selected genes in *S. horneri* and seven other brown algae species: *E. siliculosus*, *S. japonica*, *S. fusiforme*, *M. pyrifera*, *N. decipiens*, *C. okamuranus*, and *U. pinnatifida* [41]. In this analysis, the coding sequences of these species were inputted into the PosiGene pipeline. *S. horneri* and *S. fusiforme* were designated as the target species, whereas *S. fusiforme* and *E. siliculosus* served as the anchor and reference species, respectively. Genes were considered positively selected if the branch-wide test yielded false discovery rates (FDRs) of less than 0.05. JPred4 was employed for secondary structure prediction [42].

## 3. Results

### 3.1. Genome Assembly

To investigate the genomic background of *S. horneri*, a total of 229,605,305 paired-end raw reads were obtained using a library with insert lengths of 350 bp on a Hiseq X-ten platform. After pre-processing the raw reads, over 200 million paired-end reads were retained. Based on k-mer analysis, the haploid genome length of *S. horneri* was predicted to be ~413.6 MB by GenomeScope, with an estimated sequence coverage of approximately 73× and a genome repeat length of 227 Mb, and a heterozygosity of ~1.23%. Using a reference-guided de novo assembly pipeline, a draft genome with a total length of 385 Mb was assembled after filtering out candidate contaminants. About 70% of the clean reads could be realigned to the assembled genome using bowtie2.

### 3.2. Gene Prediction and Annotation

RepeatMasker identified a total length of approximately 118 Mb for repetitive sequences, accounting for approximately 30.6% of the draft genome size. After masking these repeats, de novo gene prediction was carried out using the BRAKER1 pipeline, resulting in the detection of 58,211 putative genes based on public RNA-seq data. The gene completeness was assessed, revealing that approximately 75.6% of the BUSCOs were detected at the protein level. Among the putative genes, 87.03% had hits in Nr, 52.67% had hits in Swiss-Prot, 57.96% had hits in Pfam, and 16.08% had hits in GO, as shown in Table 1. In the Nr alignment analysis, *Ectocarpus* sp. was the species with the best hit, with 16,337 genes identified.

Using eggNOG-mapper, a large number of genes (75.49%) was assigned to COG functional classifications (Figure 1). The largest cluster in the COG analyses was the “function unknown” cluster, which accounted for 18.77% of the total COG assignments. This was followed by the “Amino acid transport and metabolism” cluster, representing 8.15% of the total COG assignments, and the “Replication, recombination and repair” cluster, representing 6.36% of the total COG assignments. Among the annotated genes, a total of 4247 genes were found to have hits in the KEGG database. Furthermore, out of these annotated genes, 1407 genes were successfully mapped onto 128 enzymes within the “Metabolism” pathway category (Supplementary Table S1).

### 3.3. Putative Genes Associated with Fucoidan Biosynthesis Metabolism

**Figure 2:**
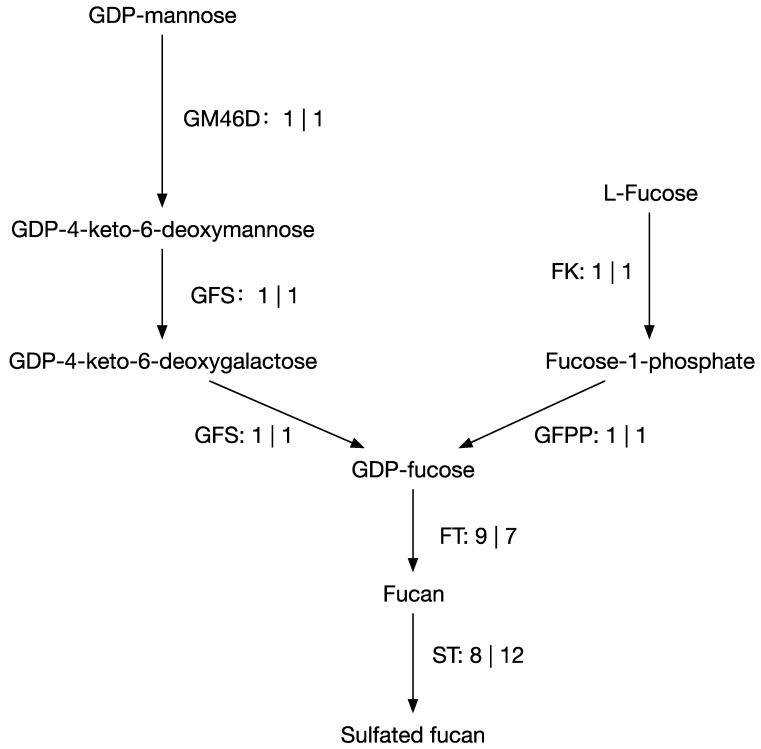
Putative genes involved in fucoidan biosynthetic pathway. GM46D: GDP-mannose 4,6-dehydratase. GFS: GDP-l-fucose synthetase. FK: L-fucokinase. GFPP: GDP-fucose pyrophosphorylase. FT: fucosyltransferase. ST: sulfotransferase. The number before and after the pipe character is the count of the corresponding enzyme in *S. fusiforme* and *S. horneri*, respectively.

The Blast search revealed the presence of genes encoding key enzymes involved in fucoidan biosynthesis metabolism in *S*. *horneri* (Figure 2). Specifically, we detected 3 genes homologous to GM46D and GFPP from *S. japonica*; 2 genes with GFS and FK from *E. siliculosus*; 7 genes matching FT from *E. siliculosus*, including 4 from GT23 and 3 from GT10; and 12 genes matching ST, including 9 from *E. siliculosus* and 3 from *S. japonica.* This differed for *S. fusiforme*, in which three genes were detected that are homologous to GM46D, GFS, and GFPP from *S. japonica*; one gene with FK from *E. siliculosus*; nine genes matching FT from *E. siliculosus*, including eight from GT23 and one from GT10; and eight genes matching ST, including seven from *E. siliculosus* and one from *S. japonica*.

### 3.4. Comparative Analysis

**Figure 3:**
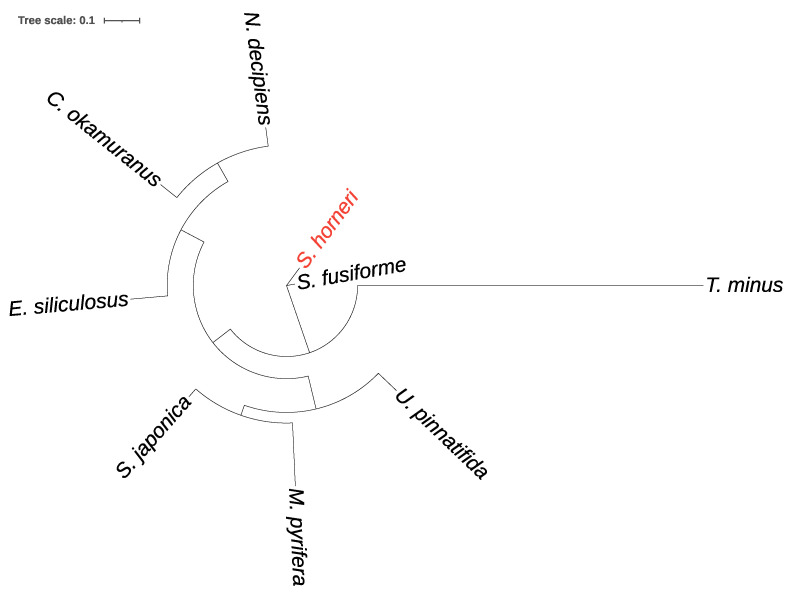
Phylogenetic tree of eight brown algae species.

In conclusion, the application of OrthoMCL resulted in the identification of a grand total of 24,453 distinct groups. Furthermore, amongst these groups, 1360 were specifically identified as one-to-one orthologs across the studied species. The sequences from these groups were then subjected to multiple sequence alignment using MAFFT and concatenated. Subsequently, a phylogenetic tree was constructed using the maximum likelihood method with RAxML (Figure 3). This tree aimed to elucidate the evolutionary relationships and genetic relatedness among the species of brown algae on a genome scale, particularly with respect to distance information. Obviously, the two species of the *Sargassum* genus cluster together with short branch lengths. Here, *T. minus* shows significant differences compared to the other species, and therefore, it was excluded from the subsequent analysis of gene family evolution.

**Figure 4:**
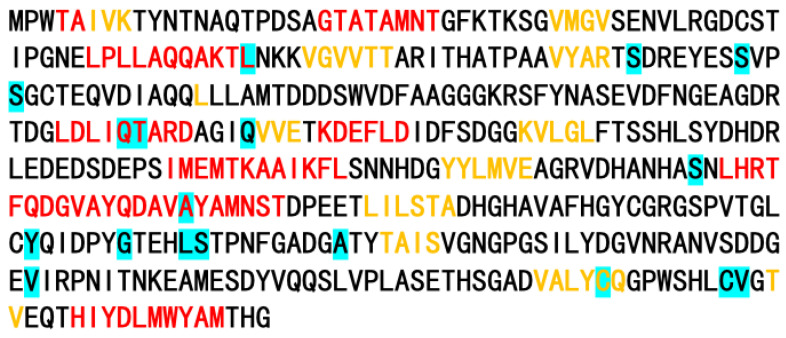
Protein secondary structure of gene g39723.t1. Helices are represented by red-colored letters, while sheets are represented by yellow-colored letters. Positive position sites are indicated by a blue-colored background.

PosiGene was employed to conduct a genome-wide search for genes that underwent positive selection in various brown algal species. By assessing the ratio of non-synonymous to synonymous substitutions, four genes exhibiting positive selection were successfully identified in *S. horneri*, with a false discovery rate (FDR) lower than 0.05 (FDR < 0.05) (Table 2). Eighteen positive selection sites were detected in the alkaline phosphatase family protein, with four located in the helix region and one in the sheet region (Figure 4).

## 4. Discussion

While a reference-based assembly can be a valuable approach for generating a high-quality genome assembly, it is important to acknowledge that errors or gaps present in the reference genome can potentially propagate to the assembled genome. Additionally, reference-based assembly runs the risk of introducing bias and overlooking crucial genomic regions that are specific to the organism under investigation. In this study, we employed the reference-based assembly method to conduct a comprehensive whole-genome survey analysis for *S. horneri*. This approach has yielded a valuable resource that will facilitate future investigations into algal genetics and genome evolution. *Sargassum* is a large genus of brown seaweed that includes over 300 species. It exhibits both dioecious and monoecious traits, making it a valuable model for studying algal evolution. The genome size of *S. horneri* was estimated to be 385 Mb, which is larger than the genome size of the *Sargassum* genus (196~319 Mb) previously estimated using static microspectrophotometry [43]. This suggests that there is high genetic diversity among the species in the *Sargassum* genus. Compared to two other well-known brown algae, the estimated genome size of *S. horneri* was larger than that of *E. siliculosus* and smaller than that of *S. japonica*. The percentage of repetitive sequences in *S. horneri* (30.6%) was slightly larger than that of *E. siliculosus* (22.7%) [14] and *S. japonica* (39%) [15], although we excluded contigs less than 200 bps from the repetitive sequence analysis, suggesting that the real repeat percentage may be higher. Interestingly, the percentage of repetitive sequence in *S. horneri* was much lower than that of *S. fusiforme* (60.7%), despite the two species having similar genome sizes (394.4 Mb). This difference in repetitive sequence content could be attributed to the inherent limitations of short-read assembly methods, which generally provide less comprehensive information on repetitive elements compared to long-read assembly methods. 

The relatively high heterozygous ratio (1.23%) can have an impact on the N50 value during the assembly of NGS reads, indicating that additional efforts, such as implementing third generation sequence technology, would be necessary to improve the quality of the genome assembly. A total of 58,211 protein-encoding genes were predicted in the assembled genome, surpassing the reported numbers for *S. fusiforme* [20], *E. siliculosus* [14], and *S. japonica* [15]. It is acknowledged that some of the identified genes may represent exons or fragments. This may be due to the short sequence length of the initial assembly, the numerous contigs, and the relatively high heterozygosity. Most of the putative genes could be aligned to known proteins from public databases with a low E-value, and nearly half of them showed the best match with *Ectocarpus* sp., indicating that most of these putative genes have been properly assigned. 

Fucoidan biosynthesis is a complex metabolic pathway involved in the production of fucoidan, a sulfated polysaccharide found in brown algae. Fucoidan is known for its diverse biological activities, including antioxidant, anticancer, anticoagulant, and immunomodulatory properties. Understanding the genes and enzymes involved in fucoidan biosynthesis is crucial for elucidating the molecular mechanisms underlying its production and biological functions. Our study focused on genes associated with fucoidan biosynthesis and compared them with known genes from other brown algae species. In the fucoidan biosynthesis pathway, *S. japonica* was found to be the best match for GM46D and GFPP, while *E. siliculosus* was the best match for GFS, FK, and FT, supporting the hypothesis that there are distinct phylogenetic sources of genes involved in the polysaccharide biosynthesis in brown algae [44,45]. Different species may have evolved distinct strategies for fucoidan biosynthesis, potentially driven by their specific ecological niches and environmental conditions. When comparing the fucosyltransferase family of *E. siliculosus* with that of the Sargassum genus, it was observed that GT65 was not present in either species. This absence of GT65 suggests the potential existence of novel fucosyltransferases or alternative enzymes in Sargassum that play a role in fucoidan biosynthesis [46]. To gain a better understanding of these enzymes and their functions, further investigations are necessary. Identifying and characterizing these enzymes could offer valuable insights into the distinct biosynthetic pathways present in Sargassum species.

The purpose of concatenating the sequences is to combine the information from multiple protein sequences, thereby increasing the accuracy and reliability of the phylogenetic analysis. By combining multiple sequences into concatenated sequences, it allows for a more comprehensive examination of the evolutionary relationships between species and provides more information to infer their genetic relationships. After concatenation, the phylogenetic tree was constructed using RAxML V8.2.3 and the PROTGAMMALG model. RAxML is a commonly used software for phylogenetic tree construction, which utilizes the maximum likelihood method to infer the tree’s topology and branch lengths. The PROTGAMMALG model is a widely used protein evolution model that considers substitution rates and types among different protein sequences to accurately estimate the phylogenetic tree. The high degree of similarity between the genomes of these two species within the genus *Sargassum* provides strong support for the utilization of the *S. fusiforme* reference-guided assembly pipeline in the genome assembly of *S. horneri.* The unicellular filamentous yellow-green algae *T. minus*, belonging to the class Xanthophyceae, are widely distributed in both freshwater and saltwater ecosystems. The genome used in this study was initially isolated from wastewater treatment ponds in San Luis Obispo, CA. It was subsequently excluded from the subsequent evolutionary analysis. In the analysis of gene family evolution, a gene encoding an alkaline phosphatase family protein was found that exhibited positive selection in *S. horneri*. Alkaline phosphatase plays an important role in utilizing dissolved organic phosphorus compounds by catalyzing the decomposition or mineralization of organic phosphorus into biologically active phosphorus in algae in subtropical coastal water [47]. Therefore, the evolution of alkaline phosphatase may improve the utilization rate of organic phosphorus and help *S. horneri* achieve population dominance, providing pivotal molecular materials for the study of the *S. horneri* golden tide.

## Figures and Tables

**Table 1:** Statistics of gene functional annotations in the *S. horneri* genome.

Database	Annotated Number (300 > Protein Length ≥ 100)	Annotated Number (Protein Length ≥ 300)	All Annotated Genes (Total)
Nr	29,413	16,859	50,634
Swiss-Prot	16,671	12,041	30,603
Pfam	18,453	13,294	33,720
GO	4859	3692	9364
COG	25,620	14,623	43,915

**Table 2:** Positively selected genes detected in *S. horneri*.

Homologous Transcript in *E. siliculosus*	Transcript in *S. horneri*	FDR	No. of Species Included	Function
Ec-21_005550.1	g21358.t1	0.022	6	Peptidyl-prolyl cis-trans isomerase, cyclophilin-type
Ec-01_007880.1	g39723.t1	0.026	6	Alkaline phosphatase family protein
Ec-12_007440.1	g8237.t1	0.026	5	Trinucleotide repeat containing 4, isoform CRA_d
Ec-14_004430.1	g8183.t1	0.026	7	Conserved unknown protein

## Data Availability

The data utilized to support the conclusions of this research are accessible from the Short Read Archive (SRA) database under the accession number PRJNA756794. The genome assemblies prior to and after decontamination have been deposited in the Figshare repository at the following URL: https://doi.org/10.6084/m9.figshare.24288274.v1 (accessed on 20 October 2023).

## Supplementary Materials

The following supporting information can be downloaded at: https://www.mdpi.com/article/10.3390/genes14101969/s1, Supplementary Table S1. Gene count of *S. horneri* mapped onto KEGG pathways.
