# Supplementary material for: The Draft Genome of the “Golden Tide” Seaweed, Sargassum horneri: Characterization and Comparative Analysis
Source: Genes (Basel). 2023 Oct 21;14(10):1969. doi: 10.3390/genes14101969 (PMC10606923; doi:10.3390/genes14101969)
Supplement: Supplementary file 1 [file genes-14-01969-s001.zip › genes-2649051-supplementary.pdf]

Supplementary Table S1. Gene count of S.horneri mapped onto KEGG pathways.

Metabolic pathway	Pathway ID	Description of pathway	Gene count
Carbohydrate metabolism	KO00010	Glycolysis / Gluconeogenesis	32
	KO00020	Citrate cycle (TCA cycle)	21
	KO00030	Pentose phosphate pathway	18
	KO00040	Pentose and glucuronate interconversions	12
	KO00051	Fructose and mannose metabolism	18
	KO00052	Galactose metabolism	12
	KO00053	Ascorbate and aldarate metabolism	18
	KO00500	Starch and sucrose metabolism	19
	KO00520	Amino sugar and nucleotide sugar metabolism	35
	KO00620	Pyruvate metabolism	31
	KO00630	Glyoxylate and dicarboxylate metabolism	26
	KO00640	Propanoate metabolism	20
	KO00650	Butanoate metabolism	13
	KO00660	C5-Branched dibasic acid metabolism	5
	KO00562	Inositol phosphate metabolism	22
Energy metabolism	KO00190	Oxidative phosphorylation	60
	KO00195	Photosynthesis	16
	KO00196	Photosynthesis - antenna proteins	6
	KO00710	Carbon fixation in photosynthetic organisms	20
	KO00720	Carbon fixation pathways in prokaryotes	16
	KO00680	Methane metabolism	18
	KO00910	Nitrogen metabolism	10
	KO00920	Sulfur metabolism	17
Lipid metabolism	KO00061	Fatty acid biosynthesis	14
	KO00062	Fatty acid elongation	8
	KO00071	Fatty acid degradation	15

	KO00072	Synthesis and degradation of ketone bodies	2
	KO00100	Steroid biosynthesis	11
	KO00140	Steroid hormone biosynthesis	3
	KO00561	Glycerolipid metabolism	21
	KO00564	Glycerophospholipid metabolism	25
	KO00565	Ether lipid metabolism	4
	KO00600	Sphingolipid metabolism	4
	KO00590	Arachidonic acid metabolism	7
	KO00591	Linoleic acid metabolism	2
	KO00592	alpha-Linolenic acid metabolism	7
	KO01040	Biosynthesis of unsaturated fatty acids	9
Nucleotide metabolism	KO00230	Purine metabolism	46
	KO00240	Pyrimidine metabolism	30
Amino acid metabolism	KO00250	Alanine, aspartate and glutamate metabolism	21
	KO00260	Glycine, serine and threonine metabolism	34
	KO00270	Cysteine and methionine metabolism	39
	KO00280	Valine, leucine and isoleucine degradation	28
	KO00290	Valine, leucine and isoleucine biosynthesis	11
	KO00300	Lysine biosynthesis	10
	KO00310	Lysine degradation	16
	KO00220	Arginine biosynthesis	19
	KO00330	Arginine and proline metabolism	22
	KO00340	Histidine metabolism	11
	KO00350	Tyrosine metabolism	13
	KO00360	Phenylalanine metabolism	10
	KO00380	Tryptophan metabolism	19
	KO00400	Phenylalanine, tyrosine and tryptophan biosynthesis	21
Metabolism of other amino acids	KO00410	beta-Alanine metabolism	12

	KO00430	Taurine and hypotaurine metabolism	3
	KO00440	Phosphonate and phosphinate metabolism	2
	KO00450	Selenocompound metabolism	14
	KO00460	Cyanoamino acid metabolism	7
	KO00471	D-Glutamine and D-glutamate metabolism	4
	KO00480	Glutathione metabolism	22
Glycan biosynthesis and metabolism	KO00510	N-Glycan biosynthesis	28
	KO00513	Various types of N-glycan biosynthesis	19
	KO00512	Mucin type O-glycan biosynthesis	1
	KO00514	Other types of O-glycan biosynthesis	2
	KO00532	Glycosaminoglycan biosynthesis - chondroitin sulfate / dermatan sulfate	1
	KO00534	Glycosaminoglycan biosynthesis - heparan sulfate / heparin	1
	KO00531	Glycosaminoglycan degradation	1
	KO00563	Glycosylphosphatidylinositol (GPI)-anchor biosynthesis	14
	KO00603	Glycosphingolipid biosynthesis - globo and isoglobo series	1
	KO00604	Glycosphingolipid biosynthesis - ganglio series	2
	KO00511	Other glycan degradation	9
	KO00540	Lipopolysaccharide biosynthesis	7
	KO00541	O-Antigen nucleotide sugar biosynthesis	7
	KO00550	Peptidoglycan biosynthesis	1
	KO00543	Exopolysaccharide biosynthesis	1
Metabolism of cofactors and vitamins	KO00730	Thiamine metabolism	9
	KO00740	Riboflavin metabolism	9
	KO00750	Vitamin B6 metabolism	8
	KO00760	Nicotinate and nicotinamide metabolism	16
	KO00770	Pantothenate and CoA biosynthesis	14
	KO00780	Biotin metabolism	8

	KO00785	Lipoic acid metabolism	14
	KO00790	Folate biosynthesis	19
	KO00670	One carbon pool by folate	11
	KO00830	Retinol metabolism	5
	KO00860	Porphyrin and chlorophyll metabolism	29
	KO00130	Ubiquinone and other terpenoid-quinone biosynthesis	16
Metabolism of terpenoids and polyketides	KO00900	Terpenoid backbone biosynthesis	27
	KO00902	Monoterpenoid biosynthesis	3
	KO00909	Sesquiterpenoid and triterpenoid biosynthesis	10
	KO00904	Diterpenoid biosynthesis	1
	KO00906	Carotenoid biosynthesis	1
	KO00981	Insect hormone biosynthesis	1
	KO00908	Zeatin biosynthesis	2
	KO00903	Limonene and pinene degradation	1
	KO00281	Geraniol degradation	1
	KO01051	Biosynthesis of ansamycins	1
	KO00523	Polyketide sugar unit biosynthesis	3
	KO01053	Biosynthesis of siderophore group nonribosomal peptides	1
Biosynthesis of other secondary metabolites	KO00940	Phenylpropanoid biosynthesis	1
	KO00901	Indole alkaloid biosynthesis	1
	KO00950	Isoquinoline alkaloid biosynthesis	4
	KO00960	Tropane, piperidine and pyridine alkaloid biosynthesis	6
	KO00232	Caffeine metabolism	1
	KO00965	Betalain biosynthesis	1
	KO00966	Glucosinolate biosynthesis	3
	KO00332	Carbapenem biosynthesis	1
	KO00261	Monobactam biosynthesis	7
	KO00521	Streptomycin biosynthesis	5

	KO00524	Neomycin, kanamycin and gentamicin biosynthesis	1
	KO00401	Novobiocin biosynthesis	1
	KO00405	Phenazine biosynthesis	2
	KO00333	Prodigiosin biosynthesis	3
	KO00254	Aflatoxin biosynthesis	1
	KO00997	Biosynthesis of various secondary metabolites - part 3	4
Xenobiotics biodegradation and metabolism	KO00362	Benzoate degradation	4
	KO00627	Aminobenzoate degradation	3
	KO00364	Fluorobenzoate degradation	1
	KO00625	Chloroalkane and chloroalkene degradation	2
	KO00361	Chlorocyclohexane and chlorobenzene degradation	1
	KO00623	Toluene degradation	1
	KO00643	Styrene degradation	5
	KO00791	Atrazine degradation	1
	KO00930	Caprolactam degradation	2
	KO00626	Naphthalene degradation	1
	KO00980	Metabolism of xenobiotics by cytochrome P450	6
	KO00982	Drug metabolism - cytochrome P450	5
	KO00983	Drug metabolism - other enzymes	17
